# Can Porphyrin–Triphenylphosphonium Conjugates Enhance the Photosensitizer Performance Toward Bacterial Strains?

**DOI:** 10.1021/acsabm.4c00659

**Published:** 2024-07-15

**Authors:** Inês Chaves, Filipe M. P. Morais, Cátia Vieira, Maria Bartolomeu, M. Amparo F. Faustino, M. Graça P. M. S. Neves, Adelaide Almeida, Nuno M. M. Moura

**Affiliations:** † CESAM, Department of Biology, University of Aveiro, Aveiro 3810-193, Portugal; ‡ LAQV-REQUIMTE, Department of Chemistry, University of Aveiro, Aveiro 3810-193, Portugal

**Keywords:** porphyrin, triphenylphosphonium, antimicrobial photodynamic therapy, photosensitizers, bacteria

## Abstract

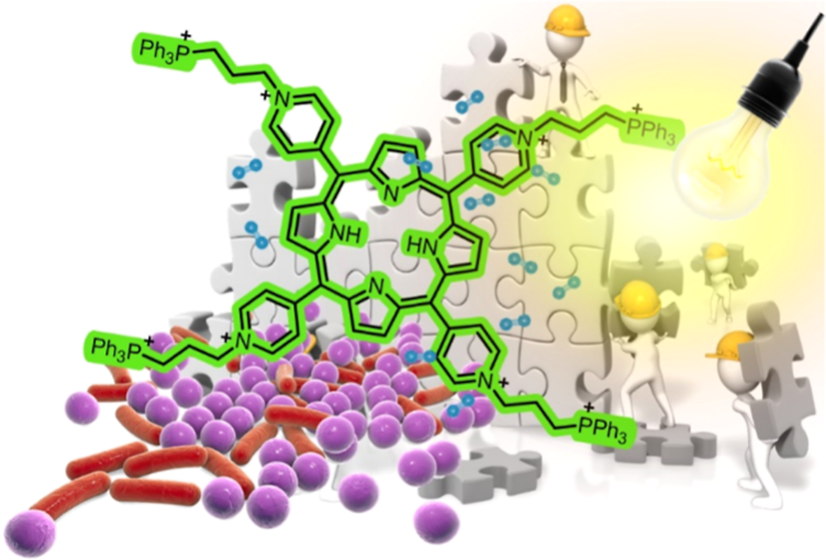

Antimicrobial photodynamic treatment (aPDT) offers an alternative option for combating microbial pathogens, and in this way, addressing the challenges of growing antimicrobial resistance. In this promising and effective approach, cationic porphyrins and related macrocycles have emerged as leading photosensitizers (PS) for aPDT. In general, their preparation occurs via *N*-alkylation of nitrogen-based moieties with alkyl halides, which limits the ability to fine-tune the features of porphyrin-based PS. Herein, is reported that the conjugation of porphyrin macrocycles with triphenylphosphonium units created a series of effective cationic porphyrin-based PS for aPDT. The presence of positive charges at both the porphyrin macrocycle and triphenylphosphonium moieties significantly enhances the photodynamic activity of porphyrin-based PS against both Gram-positive and Gram-negative bacterial strains. Moreover, bacterial photoinactivation is achieved with a notable reduction in irradiation time, exceeding 50%, compared to 5,10,15,20-tetrakis(1-methylpyridinium-4-yl)porphyrin (**TMPyP**), used as the reference and known as good PS. The improved capability of the porphyrin macrocycle to generate singlet oxygen combined with the enhanced membrane interaction promoted by the presence of triphenylphosphonium moieties represents a promising approach to developing porphyrin-based PS with enhanced photosensitizing activity.

## 1 Introduction

Antimicrobial resistance poses a global health threat, with the potential to impact not only human life but also other sectors, including healthcare, veterinary, and agriculture.^1−4^ Factors such as inadequate access to clean water, sanitation, insufficient infection prevention and control measures, and the improper use of antimicrobials contribute to the proliferation of drug-resistant pathogens.^5−9^ Therefore, there is a pressing need to prioritize the development of novel, effective, and affordable antimicrobial therapeutics.

Antimicrobial photodynamic treatment (aPDT) has garnered significant attention from the scientific community due to its achievements in both clinical and environmental settings.^1,10−12^ The aPDT approach relies on the activation of a nontoxic agent, referred to as photosensitizer (PS), using harmless visible light in the presence of dioxygen (^3^O_2_), thereby generating highly cytotoxic reactive oxygen species (ROS), particularly singlet oxygen (^1^O_2_). These ROS can effectively oxidize various cellular components, swiftly rendering the cells inactive.^2,7,13−15^ aPDT offers several advantages compared to traditional antimicrobials. First, the multitarget nature of aPDT, responsible for the elimination of microbial organisms, reduces the chances of resistance development.^16^ Additionally, the treatment remains efficient regardless of the microbial antibiotic resistance profile.^6,8,17,18^ Moreover, the photodynamic approach can be successfully used to combat bacteria but also viruses, fungi and parasites.^10,19^

The efficacy of photodynamic action is significantly related on the structure of the PS and its ability to produce ROS. While various classes of organic compounds have been explored as PS in aPDT, porphyrins and related macrocycles stand out as prime candidates.^2,8,20−23^ The considerable focus on synthesizing porphyrinoids, recognized for their biological relevance, is due to their unique properties suitable for a broad range of applications.^24−30^

In the context of PS for aPDT, porphyrinoids exhibit distinctive characteristics that set them apart. These include chemical versatility, photo- and storage stability, strong absorption in the visible region, efficient photoinduced reactions with dioxygen, low toxicity, and high binding affinity to cellular components such as membranes, proteins, or nucleic acids.^10,31^

The cell wall structure of Gram-positive and Gram-negative bacteria plays a significant role in their sensitivity to photodynamic inactivation. Generally, all classes of PS molecules efficiently penetrate/bind to Gram-positive bacteria, rendering them inactive. However, Gram-negative bacteria are often more resistant to the aPDT mediated by a PS due to the presence of an additional outer membrane in the bacteria cell.^32,33^ However, cationic PS have been recognized to improve the susceptibility of Gram-negative bacteria toward aPDT; this is ascribed to the strong electrostatic interactions involving the negatively charged lipopolysaccharides of the bacteria outer membrane and the PS positive charges.^34,35^

Among the most extensively studied and effective PS in aPDT are 5,10,15,20-tetrakis(1-methylpyridinium-4-yl)porphyrin (**TMPyP**) and its analogs, particularly for the photoinactivation of both Gram-positive and Gram-negative bacteria.^36,37^ Although the presence of positive charges in the porphyrinoids structure is notably advantageous for aPDT efficacy,^38,39^ the strategy of developing cationic porphyrin-based PS is somewhat limited. In general, the approaches involve the quaternization of nitrogen atoms from pyridyl or amino moieties at *meso*-positions. This fact restricts the modulation of the PS photochemical and photophysical properties and also its degree of hydrophilicity through adjustments in the number of cationic moieties and/or the introduction of alkyl chains of varying lengths onto the nitrogen atoms.^34,40^

Other strategies imply the use of laborious and complex synthetic routes to prepare natural porphyrin analogs or the use of other strategies to induce the aPDT effect toward Gram-negative bacteria, such as the use of membrane disorganizing agents or the expensive attachment of cationic polypeptide to neutral or anionic PS.^18,40^

Triphenylphosphonium-based compounds are widely recognized for their specificity in targeting mitochondria, making them valuable for both antitumoral treatments and diagnostic purposes.^41^ Their notable attributes, including a large hydrophobic surface area and distributed charge, allow for high affinity to the lipid membranes of microorganisms, particularly bacteria, rendering them potent antibacterial agents.^42−44^ These compounds primarily function by disrupting the cell membrane and inhibiting multidrug efflux pumps.^42^ However, small molecules containing triphenylphosphonium moieties exhibit certain drawbacks, such as poor heat resistance and persistence, making them susceptible to efficacy loss during use and posing some toxicity concerns. One approach to mitigate these issues involves the preparation of polymers containing triphenylphosphonium units. Nonetheless, this strategy may lead to heterogeneity, and high-molecular-weight polymers can lose their activity due to entanglement and chain obstruction.^44^

To date, only one example of triphenylphosphonium-based compounds has been reported for use in blue light therapy to photoinactivate the Gram-positive bacteria *Staphylococcus aureus* and *Enterococcus faecalis*, although with photoinactivation inefficiency against Gram-negative *Escherichia coli*.^45^

Regarding the preparation of porphyrin–triphenylphosphonium derivatives, this area appears largely unexplored based on current knowledge. Few examples have been reported in the literature. For instance, Officer and co-workers^46^ described the preparation of β-modified porphyrin–triphenylphosphonium as a template for Wittig reactions, while Haber et al.^47^ utilized the trihexyl(tetradecyl)phosphonium cation as a counterion for an anionic porphyrin. Additionally, Král and co-workers^48^ reported the synthesis of the first *meso*-tetraarylporphyrin bearing four triphenylphosphonium moieties, which was used by the same group for DNA binding^49^ and later by García and co-workers to prepare porphyrin-doped CdTe quantum dots.^50^ Lei et al. described the use of a porphyrin bearing one triphenylphosphonium moiety in photodynamic therapy against MCF-7 cancer cells.^51^ However, the utilization of porphyrin–triphenylphosphonium-based PS in the photodynamic inactivation of microorganisms remains largely unexplored.

**Figure 1:**
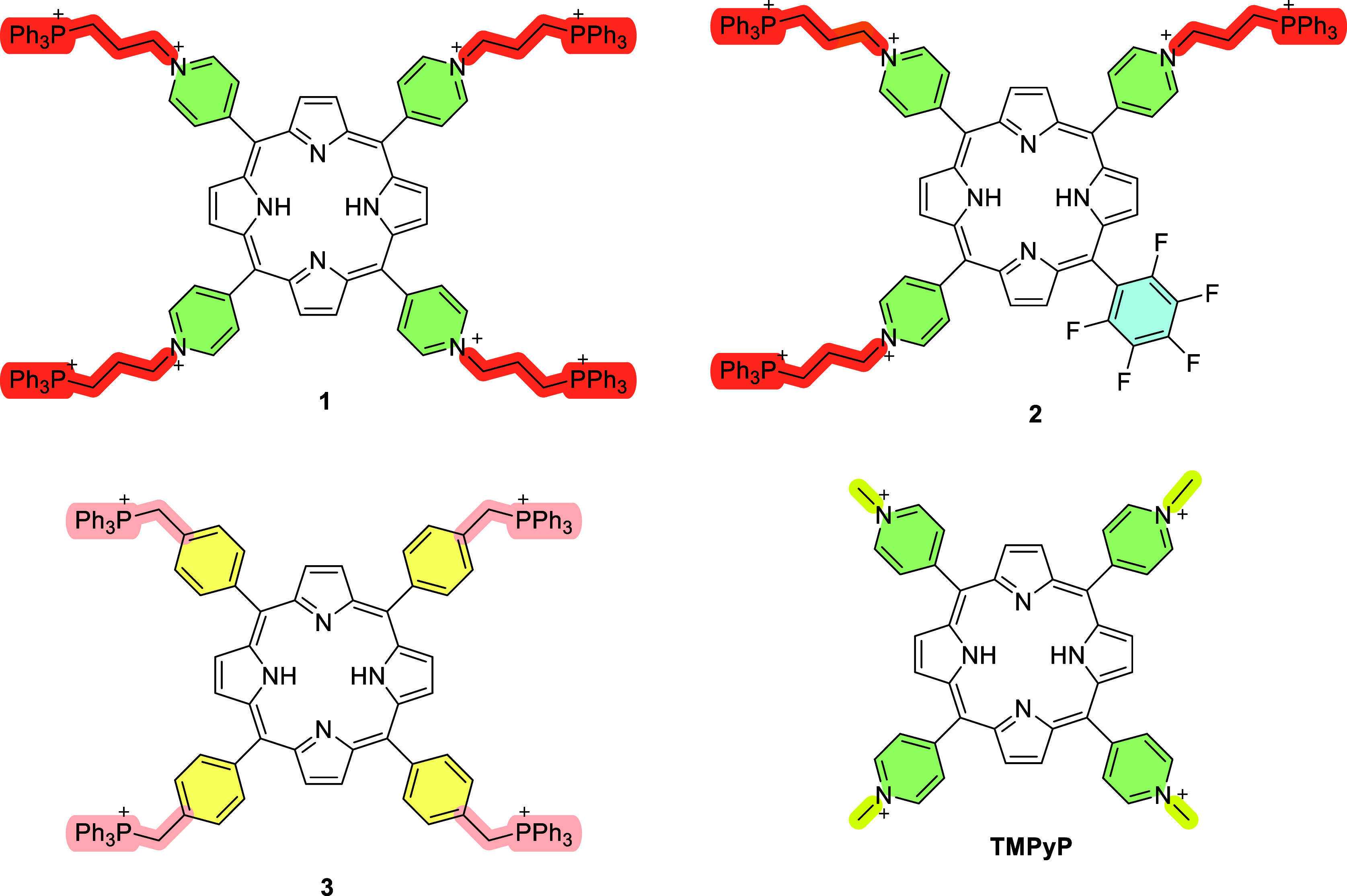
Structures of the porphyrin–triphenylphosphonium derivatives **1–3** and of the reference **TMPyP**.

Therefore, this research work aimed to evaluate the impact of triphenylphosphonium moieties in the photodynamic microbial ability of porphyrins **1**, **2**, and **3** containing eight, six and four positive charges, respectively (Figure 1) toward Gram-positive and Gram-negative bacterial strains. It was envisaged that the presence of a large hydrophobic surface area and a distributed high number of positive charges due to the triphenylphosphonium units could enable a better adhesion of the PS to biological structures, enhancing the photodynamic activity against both Gram-negative and Gram-positive bacteria. To corroborate the results obtained, the (photo)stability, the ability to generate singlet oxygen and the photodynamic efficiency of the selected conjugates were compared to that of the well-studied tetracationic porphyrin derivative, **TMPyP**, used as reference.

## 2 Materials and Methods

### 2.1 Generalities

Nuclear magnetic resonance (NMR) spectra were recorded on a Bruker AVANCE 300 or Bruker AVANCE 500 spectrometers at 300.13 and 125.76 MHz for ^1^H and ^13^C NMR, respectively. DMSo-*d*_6_ was used as solvent and tetramethylsilane as internal reference. The UV–vis spectra were recorded on an UV-2501 PC Shimadzu spectrophotometer using DMF as solvent.

### 2.2 Synthesis

#### 2.2.1 Synthesis of Porphyrin–Triphenylphosphonium Derivatives **1** and **2**

To a solution of **TPyP** (30 mg, 3.24 × 10^–5^ mol) or **TriPyPF** (30 mg, 3.24 × 10^–5^ mol) in DMF (1.5 mL) was added an excess of equivalents of (3-bromopropyl)triphenylphosphonium bromide per pyridyl units (16 or 12 equiv) and the reaction mixture was kept under magnetic stirring at 80 °C for 15 h. After this period, the reaction was cooled to room temperature and then it was added diethyl ether. The obtained precipitate was filtrated and washed with toluene. The solid was purified by column chromatography, using aluminum oxide 90 active neutral as the stationary phase and a CH_2_Cl_2_/MeOH mixture (97:3 and 80:20) as the eluent. After crystallization in a CH_2_Cl_2_/hexane mixture, porphyrin–triphenylphosphonium conjugates **1** and **2** were obtained in 97 and 94%, respectively (Figures S1–S6).

##### 2.2.1.1 Compound **1**

^1^H NMR (300 MHz, DMSO-*d*_6_): δ 9.77 (8H, d, *J* = 6.9 Hz, H-*o*-Py), 9.35 (8H, s, H-β), 9.04 (8H, d, *J* = 6.9 Hz, H-*m*-Py), 7.96–8.06 (24H, m, H-*o*-Ph), 7.77–7.90 (36H, m, H-*m,p*-Ph), 5.41–5.21 (8H, m, –CH_2_), 4.34–4.25 (8H, m, –CH_2_), 2.72–2.62 (8H, m, –CH_2_), −3.11 (2H, s, H-*N*) ppm. ^13^C NMR (125 MHz, DMSO-*d*_6_): δ 163.1, 157.2, 144.1, 135.8, 134.2, 134.2, 134.0, 133.9, 133.1, 131.0, 130.9, 130.8, 130.7, 118.8, 118.1, 116.3, 36.4, 31.3, 24.4. ppm. HRMS-ESI(+): *m*/*z* calcd for C_124_H_110_N_8_P_4_ 229.34700 M^8+^; found, 229.35739.

##### 2.2.1.2 Compound **2**

^1^H NMR (300 MHz, DMSO-*d*_6_): δ 9.84–9.66 (6H, m, H-*o*-Py), 9.51–9.18 (8H, m, H-β), 9.10–8.98 (8H, m, H-*m*-Py), 8.13–7.93 (27H, m, H-*o*-Ph), 7.92–7.73 (18H, m, H-*m,p*-Ph), 5.41–5.21 (6H, m, –CH_2_), 4.35–4.17 (6H, m, –CH_2_), 2.71–2.59 (6H, m, –CH_2_), −3.13 (2H, s, H-*N*) ppm. ^13^C NMR (125 MHz, DMSO-*d*_6_): δ 157.2, 144.2, 135.7, 134.4, 134.31, 133.14, 133.07, 131.0, 130.9, 119.0, 119.0, 118.4, 118.3, 116.7, 116.4, 103.5, 70.2, 60.3, 24.4 ppm. HRMS-ESI(+): *m*/*z* calcd for C_104_H_85_F_5_N_7_P_3_ 269.92776 M^6+^; found, 269.93333.

#### 2.2.2 Synthesis of Porphyrin–Triphenylphosphonium Derivative **3** and of **TMPyP**

Compounds **3** and **TMPyP** were synthesized using previous procedures.^48,52^^1^H NMR and UV–vis spectroscopy, and mass spectrometry were used to confirm the structures of both porphyrin-based PS. The spectroscopic results are in good agreement with the published results (Figures S7–S10).

##### 2.2.2.1 Compound **3**

^1^H NMR (300 MHz, DMSO-*d*_6_): δ 8.82 (8H, m, H-β), 8.04–7.91 (60H, m, PPh_3_), 8.05–7.98 (8H, m, H-*o,m*-Ph), 5.35–5.21 (8H, m, –CH_2_), −3.12 (2H, s, H-*N*) ppm. MS-ESI(+): *m*/*z* calcd for C_120_H_94_N_4_P_4_ 428.9 M^4+^; found, 429.1.

##### 2.2.2.2 TMPyP

^1^H NMR (300 MHz, DMSO-*d*_6_): δ 9.48 (8H, d, *J* = 6.7 Hz, H-*o*-Py), 9.22 (8H, s, H-β), 9.00 (8H, d, *J* = 6.7 Hz, H-*m*-Py), 4.74 (12H, s, *N*CH_3_), −3.10 (2H, s, H-*N*) ppm. MS-ESI(+): *m*/*z* calcd for C_44_H_38_N_8_ 169.58 M^4+^; found, 169.57.

### 2.3 Stock Solutions of the PSs

Stock solutions of each PS **1–3** and **TMPyP** were prepared in dimethylformamide (DMF) for singlet oxygen generation assays or dimethyl sulfoxide (DMSO) for biological and (photo)stability assays, at concentrations of 100 or 500 μM, respectively. These solutions were stored in the dark at room temperature. Prior to each assay, the PS solutions were sonicated for 30 min at room temperature to ensure uniformity and dispersion of the stock solutions (using an ultrasonic bath, Nahita 0.6 L, 40 kHz).

### 2.4 Absorption and Emission Spectra

UV–vis absorption spectra were recorded in PBS at a concentration of 5.0 μM using a UV-2501PC Shimadzu spectrophotometer (Shimadzu Corporation, Kyoto, Japan). Emission spectra of the studied compounds in PBS were obtained with a fluorimeter (FluoroMax Plus; HORIBA Scientific, Piscataway, NJ, USA) with excitation at the Soret band maximum for each compound (see Table 1) and a 5 nm slit width.

**Table 1:** Absorption and Emission Data of Compounds **1–3** and **TMPyP** in PBS Solution

compd	soret band (nm)	log ε	Q bands (nm)	log ε	λ_em_ (nm)
**1**	428	5.17	520	4.09	710
			556	3.76	
			586	3.75	
			642	3.22	
**2**	423	5.09	516	4.11	664
			551	3.61	708
			583	3.69	
			634	3.17	
**3**	429	3.91	521	3.24	649
			557	3.12	714
			589	2.91	
			645	2.87	
**TMPyP**	423	5.36	518	4.16	706
			552	3.70	
			585	3.77	
			640	3.10	

### 2.5 Dark and Photostability

Diluted solutions of each PS at 5.0 μM in PBS were prepared from 500 μM stock solutions for dark stability tests. These solutions were kept in the dark with magnetic agitation at room temperature throughout the assay. Absorption spectra were then recorded at 0, 5, 10, 15, and 30 min using a HALO DB-20 Dynamica spectrophotometer (Dynamica, Livingston, UK) within the 350–700 nm range. For photostability assays, glass cuvettes containing the solutions were irradiated with white light PAR (380–700 nm) from a 30 W, 2000 lm LED system (LUMECO) at an irradiance of 25 mW cm^–2^ for 30 min. Absorption spectra were recorded at 0, 5, 10, 15, and 30 min after irradiation using the HALO DB-20 Dynamica spectrophotometer.

### 2.6 Singlet Oxygen Generation

A 10.0 mM stock solution of 1,3-diphenylisobenzofuran (DPiBF) was prepared in DMF. Solutions (2.5 mL) were then prepared in quartz cells containing DPiBF at a concentration of 50 μM and one of the PS, **1**, **2**, **3** or **TMPyP** at a concentration of 0.5 μM. The cuvettes containing the DPiBF/PS mixture (50/0.5 μM ratio) were placed under magnetic stirring at room temperature and irradiated with red light LEDs (λ = 630 ± 20 nm) at an irradiance of 11 mW cm^–2^. This allowed the monitoring of singlet oxygen production by measuring the decrease in DPiBF absorbance at 415 nm at 60 s intervals during 600 s of irradiation.

### 2.7 Biological Evaluation Studies

#### 2.7.1 Bacterial Strains and Growth Conditions

The *E. coli* strain used was genetically modified in our research group by the insertion of two plasmids (pHK724 and pHK555) into the chemically competent *E. coli* Top 10 strain (Invitrogen, Massachusetts, USA).^53^ These plasmids contain the *lux* operon including genes essential for luciferase enzyme coding and biosynthetic enzymes, required for light production, from the naturally bioluminescent marine bacterium *Aliivibrio fischeri*.^53^ Before each assay, a bacterial suspension stored at −80 °C in 10% glycerol was transferred to 25 mL of Tryptic Soy Broth (TSB; Merck KGaA, Darmstadt, Germany) and incubated at 25 °C for 18–24 h at constant agitation (120 rpm) until it reached the stationary growth phase at approximately 10^9^ colony-forming units per mL (cfu mL^–1^). The correlation between cfu mL^–1^ and the bioluminescence signal of the bacterial strain used in this work has been previously established and reported.^53^ As a Gram-positive bacterial model, it was selected the MRSA strain DSM 25693, which produces staphylococcal enterotoxins A, C, H, G and I (Leibniz Institute, DSMZ-German Collection of Microorganisms and Cell Culture GmbH, Braunschweig, Germany).^54^ This strain was maintained on Tryptic Soy Agar (TSA; Merck KGaA, Darmstadt, Germany) at 4 °C. Prior to each assay, three colonies were transferred to 25 mL of TSB culture medium and incubated at 37 °C for 18–24 h with agitation (120 rpm). Subsequently, 250 μL of the previous culture were transferred to 25 mL of fresh TSB medium and incubated under the same conditions to promote bacterial growth to the stationary phase (approximately 10^9^ cfu mL^–1^).

#### 2.7.2 Cellular Adsorption of PSs

Bacterial suspensions were prepared in PBS (approximately 10^7^ cfu mL^–1^) in the presence of each PS studied (**1–3** and **TMPyP**), with a final volume of 2 mL. The suspensions were incubated in the dark with magnetic stirring for 15 min at room temperature to allow PS binding to bacterial cells. The studies were conducted at the highest selected PS concentrations for photodynamic inactivation assays (1.0 and 5.0 μM for *S. aureus* and *E. coli*, respectively). Following the incubation in the dark, 1 mL of each control and sample was centrifuged for 5 min at 10,000 rpm (1730R, Gyrozen Co., Ltd., Gimpo, South Korea). The unbound PS in the bacterial cells, present in the supernatant, was removed, and the bacterial pellet was washed three times with PBS and centrifuged under the same conditions. The washed pellets were resuspended in 500 μL of DMSO, vigorously vortexed, sonicated for 30 min, and vortexed again to disrupt the bacterial cells. The contents were transferred to dark 96-well microplates, and the fluorescence of PS bound to the cells was assessed by fluorescence spectroscopy using a spectrofluorometer (FluoroMax-3; HORIBA Scientific, Piscataway, NJ, USA) with a 2 nm slit width. All compounds were excited at a wavelength of 429 nm and emission spectra collected between 550 and 850 nm. The fluorescence intensity measured in the samples allowed the determination of the concentration of PS bound to the cells through interpolation using a calibration curve constructed from known concentrations of each PS. Simultaneously, 1 mL aliquots of the samples and controls were collected after the dark incubation period to determine the concentration of viable cells by the drop-seeding method.^55^ For this, the collected aliquots were serially diluted in PBS, and then 10 μL of each sample was surface seeded on TSA and incubated for 24 h at 37 °C. After this period, bacterial colonies were counted in the most appropriate dilution to determine cfu mL^–1^. The analyses were performed in duplicate, with two replicates per assay (*n* = 4). The results were expressed in nM PS/log cfu mL^–1^.

#### 2.7.3 Antimicrobial Photodynamic Therapy Assays

In each assay, a bacterial suspension in the exponential growth phase was diluted (1:100) in PBS and distributed in 6-well plates. Subsequently, an appropriate volume of each PS was added to achieve the desired concentration, specifically 1.0 and 5.0 μM for *E. coli* (porphyrin **3** was also tested at a concentration of 10.0 μM), and 0.1 and 0.5 μM for *S. aureus*. These concentrations were chosen considering the lower susceptibility of Gram-negative bacteria to aPDT compared to Gram-positive ones.^55,56^ Simultaneously, light controls (LC) containing a bacterial suspension in PBS exposed to light and, dark controls (DC) containing a bacterial suspension incubated with each PS kept in the dark throughout the treatment were prepared. After preparation, the samples and controls were incubated in the dark for 15 min with agitation (120 rpm) to promote PS binding to bacterial cells. Subsequently, the samples and LC were irradiated with white light (25 mW cm^–2^) for 90 min, while the DC were kept in the dark. To determine bacterial viability, 700 μL aliquots for *E. coli* and 150 μL aliquots for *S. aureus* were collected immediately after the dark incubation period (0 min) and after 15, 30, 45, 60, and 90 min of white light irradiation (25 mW cm^–2^, LED LUMECO, 30 W). In the case of *S. aureus*, the collected aliquots were drop-seeded as described earlier, and the concentration of viable cells was expressed as log cfu mL^–1^. For *E. coli*, the bioluminescence signal was measured using a luminometer (reading range: 300–650 nm; peak wavelength of 420 nm) (TD-20/20 Luminometer, Turner Designs, Inc., USA) and expressed as log URL. For each condition tested, at least three independent assays were performed, each with two replicates.

#### 2.7.4 Statistical Analysis

Statistical analysis was conducted using GraphPad Prism 8. Normal distribution was verified using the Kolmogorov–Smirnov test, and variance homogeneity was evaluated with the Brown–Forsythe test. For PS cell adhesion assessment assays, differences in the concentration of each PS bound to bacterial cells were assessed through one-factor ANOVA and Tukey’s multiple comparison tests. In the case of photodynamic inactivation biological assays, differences in bacterial concentration in samples and controls were evaluated at each time point through two-factor ANOVA and Tukey’s multiple comparison tests. Values with *p* < 0.05 were considered significant.

## 3 Results and Discussion

### 3.1 PSs Synthesis and Characterization

**Scheme 1:**
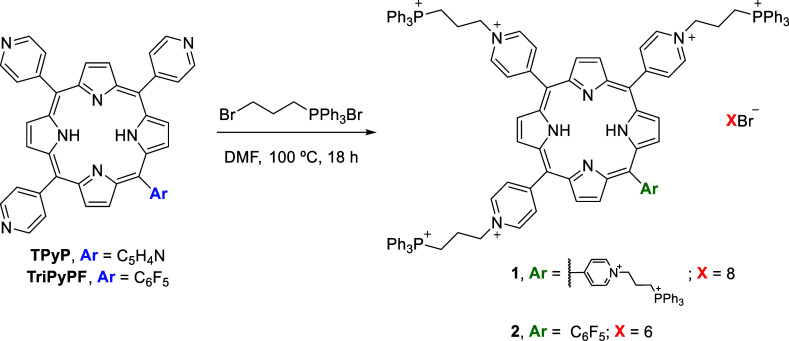
Synthetic Approach to Synthesize Porphyrin–Triphenylphosphonium Conjugates **1** and **2**

The synthesis of the octacationic porphyrin–triphenylphosphonium conjugate **1** was carried out by reacting 5,10,15,20-tetra(pyrid-4-yl)porphyrin (**TPyP**) with (3-bromopropyl)triphenylphosphonium bromide in *N*,*N*-dimethylformamide (DMF). After 18 h the *N*-alkylation of the *meso* pyridyl units with the triphenylphosphonium salt afforded the expected porphyrin–triphenylphosphonium derivative **1** in 97% yield. The asymmetric porphyrin–triphenylphosphonium derivative **2** was isolated in 94% yield from 5,10,15-tris(pyrid-4-yl)-20-(pentafluorophenyl)porphyrin (**TriPyPF**) by using an analogous synthetic approach (Scheme 1).

The tetracationic porphyrin derivatives **3** and **TMPyP** were both prepared following well-established protocols and previously reported in the literature.^48,52^ The porphyrin-triphenylphosphoinum derivative **3** was synthesized through the reaction of *meso*-tetrakis(bromo-*p*-tolyl)porphyrin with triphenylphosphine, while the synthesis of **TMPyP** involved the *N*-alkylation of pyridyl units of **TPyP** with methyl iodide.

Considering the target application, the absorption and emission spectra of all PS studied were recorded in PBS at 298 K (Table 1).

**Figure 2:**
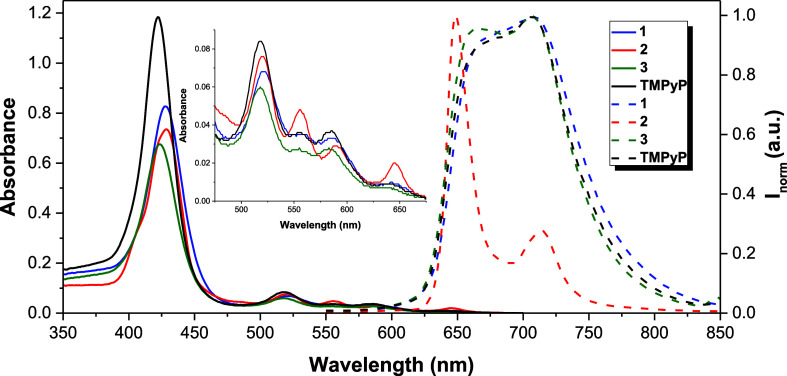
Absorption and normalized emission spectra of porphyrin–triphenylphosphonium conjugates **1–3** and **TMPyP** in PBS at 298 K (λ_exc**1**_ = 428 nm; λ_exc**2**_ = λ_exc**TMPyP**_ = 423 nm; λ_exc**3**_ = 429 nm). The figure inset represents the absorption at the Q bands region.

Typical absorption spectra of free base *meso*-tetraarylporphyrins with a *D*_2*h*_ (**1**, **3** and **TMPyP**) and *C*_2*v*_ (**2**) symmetry were provided by the PS studied (Figure 2). For compounds **1** and **3**, the Soret band, attributed to π–π* transitions from the ground state to the second excited state (S_0_ → S_2_), was red-shifted by 5 and 6 nm, respectively, in comparison with compound **2** and **TMPyP**. Regarding the Q bands region, ascribed to the weak π–π* transitions from S_0_ → S_1_, all the PS displayed four bands ranging from 516 to 645 nm. In general, there are no induced significant changes in UV–vis spectra due to the incorporated triphenylphosphonium salt moieties at porphyrin *meso* positions. However, a decrease in the intensity of the Soret band for the porphyrin–triphenylphosphonium derivatives **1–3** has been observed.

The PS containing pyridinium units **1**, **2** and **TMPyP** display analog emission spectra with an enlarged emission band with a maximum around 710 nm and a shoulder band at around 650 nm, probably due to the partial overlapping of the two canonical peaks. The emission spectrum of compound **3** exhibits an intense emission band at 649 nm and a week at 714 nm. This is a typical emission spectrum of *meso*-tetraarylporphyrins with a nearly unchanged vibronic state upon excitation, being the emission bands ascribed to Q_*x*_ (0–0) and Q_*x*_ (0–1) transitions.

Among other factors, the photodynamic performance of porphyrin-based PS can be significantly influenced by how stable they are in a solution, whether in the absence or presence of light. In line with this, the behavior of each porphyrin derivative was examined in PBS, by monitoring their absorption at the Soret band maximum over a 30 min period. These assays were performed both in the dark and under the same irradiation conditions as in the biological experiments. The results are summarized in Table 2.

**Table 2:** Photostability of Porphyrin-Based PS **1–3** and **TMPyP** at 5.0 μM, after Irradiation with White Light at an Irradiance of 25 mW cm^–2^ for Different Periods of Irradiation (0–30 min)^a^

conditions	PS	λ_max_ (nm)	irradiation time (min)				
			0	5 (%)	10 (%)	15 (%)	30 (%)
dark	**1**	428	0	4	5	6	10
	**2**	423	0	2	5	6	8
	**3**	429	0	13	15	22	25
	**TMPyP**	423	0	1	1	1	1
light	**1**	428	0	4	7	9	13
	**2**	423	0	6	9	12	20
	**3**	429	0	25	30	35	35
	**TMPyP**	423	0	2	3	4	6

a Results expressed as absorption decay percentage calculated by the ratio of absorbance at λ_max_ at different periods of time and absorbance before irradiation.

In general, under dark conditions, the porphyrin–triphenylphosphonium derivatives **1** and **2** exhibited relatively good stability (absorption decays of 10 and 8%, respectively) and, consequently, high solubility in PBS. On the other hand, porphyrin **3** showed an absorption decrease of 25% after 30 min, due to its tendency to aggregate and form molecular clusters. The reference porphyrin-based PS, **TMPyP**, exhibited as expected the highest stability, during the assay with no aggregation tendency under the tested conditions.

Under light irradiation conditions (white light at an irradiance 25 mW cm^–2^), and considering the absorption profile under dark conditions, the porphyrin–triphenylphosphonium derivative **1** demonstrated to be the most photostable PS, after being exposed to white light for 30 min (3% decay), with similar photostability behavior of the photostable **TMPyP** (5% decay). Under the same conditions, compounds **2** and **3** exhibited a photodecay of 12 and 10%, respectively. These results allowed to conclude that PS **1** show adequate (photo)stability features to be used in photodynamic processes, while PS **2** and **3** its tendency to aggregate in PBS, namely derivative **3** can somehow limit its aPDT efficiency.

The scientific community assumes that the type II mechanism is the most relevant one in aPDT mediated by porphyrin-type PS. So, it is crucial to assess the ability of these compounds to generate ROS, particularly ^1^O_2_, as it is the key cytotoxic species involved in aPDT action occurring via type II mechanism.

In line with this, was evaluated the effectiveness of porphyrin–triphenylphosphonium conjugates **1–3** and **TMPyP** in generating ^1^O_2_ using an indirect method based on the photo-oxidation of 1,3-diphenylbenzofuran (DPiBF). This chemical scavenger, with a maximum absorption at 415 nm, reacts with ^1^O_2_ in a [4 + 2] cycloaddition process, affording the colorless 1,2-dibenzoylbenzene.^57^

**Figure 3:**
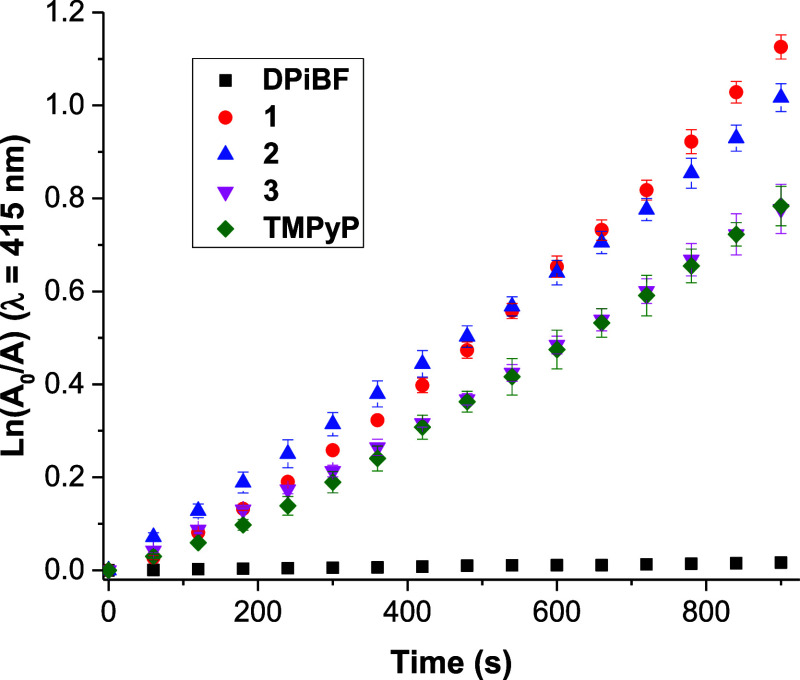
Evaluation of the decomposition of DPiBF (50 μM) in the presence and in the absence of each porphyrin at a concentration of 0.5 μM in DMF, under irradiation with red light (630 ± 20 nm).

The results summarized Figure 3 show that all the PS **1–3** were capable of producing ^1^O_2_. The octacationic PS **1** presented the highest capacity to generate this cytotoxic species, followed by the hexacationic PS **2** and then by the tetracationic porphyrin-base derivatives **3** and **TMPyP**. The phosphonium derivatives **1** and **2** showed DPiBF decay rates close to each other and 30% higher to that exhibited by PS **3** and **TMPyP**. Among porphyrin–triphenylphosphonium-based PS, the lower ^1^O_2_ producer was PS **3**. The lower ^1^O_2_ production might be associated with its higher tendency to aggregate.

It is worth to notice that after a 900 s irradiation assay, was not observed a noticeable decrease in the DPiBF absorption in the control sample, conducted in the absence of PS. This demonstrates that the DPiBF decomposition only occurs when the solution is irradiated in the presence of the studied PS, confirming their crucial role in generating ^1^O_2_.

### 3.2 aPDT Assays

The promising photophysical and photochemical features of the porphyrin–triphenylphosphonium conjugates **1–3** prompted us to evaluate their photodynamic activity toward bacterial strains. The aPDT assays were carried out using methicillin-resistant *S. aureus* (MRSA) and *E. coli*, as Gram-positive and Gram-negative bacterial models, respectively. These models are often used in aPDT studies and recognized for their disparate antimicrobial susceptibility, caused by the differences in their cell wall structures.^58,59^ Furthermore, both strains are clinically relevant, being primary etiological agents in a wide range of diseases, including bloodstream infections, and are often resistant to multiple antimicrobials.^60^ The evaluation of triphenylphosphonium conjugates’ activity also included **TMPyP** as a reference PS. **TMPyP** is the most studied porphyrin-based PS and as revealed to be highly efficient in the photoinactivation of both Gram-positive and Gram-negative bacteria among other microorganisms.^61^

#### 3.2.1 Bacterial Uptake

**Figure 4:**
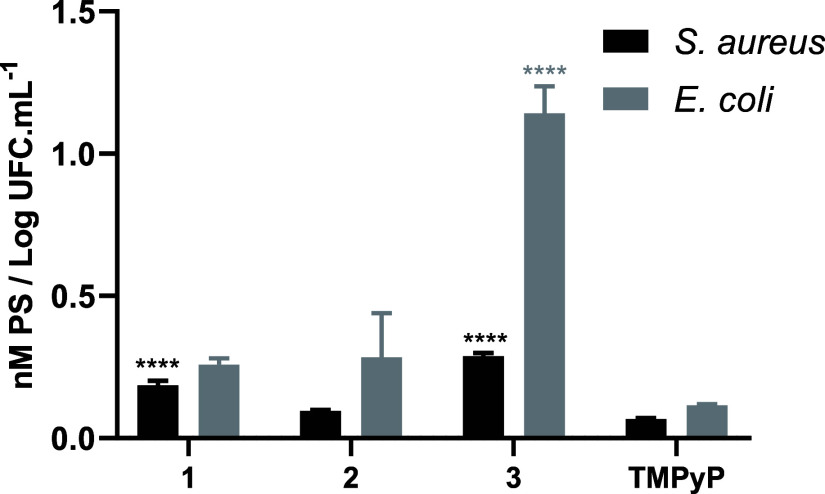
Cellular adhesion of porphyrin–triphenylphosphonium derivatives **1–3** and **TMPyP** to *S. aureus* (black) and*E. coli* (gray), at the concentrations of 0.5 and 5.0 μM, respectively. The values presented correspond to two independent assays with two replicates each; error bars express the standard deviation value. Statistically significant differences (*p* < 0.05) between PS **1–3** and **TMPyP** are marked by (****).

The affinity of PS for bacterial cells is a crucial property for aPDT efficiency. Accordingly, the ability of each PS to adhere to bacterial structures was evaluated. The assays were conducted on both MRSA and *E. coli* at the highest concentrations used in the biological tests, 0.5 and 5.0 μM, respectively. The results obtained are represented in Figure 4.

For both bacterial models, the porphyrin–triphenylphosphonium derivatives exhibited, in general, higher bacterial cell adhesion values than **TMPyP**.

In *S. aureus*, PS **3** exhibited the highest adhesion capacity (0.22 nM PS/log cfu mL^–1^, *p* < 0.05), followed by PS **1** (0.12 nM PS/log cfu mL^–1^, *p* < 0.05) and then by PS **2** (0.03 nM PS/log cfu mL^–1^, *p* < 0.05). In *E. coli*, all compounds showed higher adsorption values than **TMPyP**, being particularly significant the great adhesion observed for compound **3** (1.03 nM PS/log cfu mL^–1^, *p* < 0.05) (Figure 4).

The adhesion values of PS **1** and PS **2** to *E. coli* cells do not differ significantly (*p* > 0.05). While an increased number of charges typically enhances the PS affinity for bacterial cells, the lipophilic or hydrophilic character of PS also plays a crucial role in their cellular binding and effectiveness in aPDT.^62,63^ Hydrophobicity is particularly advantageous as it promotes effective solubilization of PS in the bilayer membrane. On the other hand, high hydrophilicity can hinder PS uptake to the cell membrane. Therefore, despite PS **1** having more charges, which theoretically enhances its affinity for *E. coli* cells, its higher hydrophilicity may counteract this effect, resulting in similar adhesion capacities between PS **1** and PS **2**. Analogous results were also observed in a previous study by our research group, where a tricationic and a tetracationic porphyrin exhibited comparable adhesion capacities.^63^ This similarity can be attributed to the more amphiphilic nature of PS **2**, which enhances their solubility within the bilayer membrane compared to the more hydrophilic octacationic porphyrin derivative **1**. The greater adhesion value obtained for derivative **3**, namely for *E. coli*, is probably related with the formation of aggregates due to its lower hydrophilicity.

#### 3.2.2 Photodynamic Inactivation of *S. aureus*

The effectiveness of porphyrin–triphenylphosphonium conjugates **1–3** and **TMPyP** in the photoinactivation of MRSA was assessed over 90 min using white light (380–700 nm) at an irradiance of 25 mW cm^–2^. The tests were conducted with PS concentrations of 0.1 and 0.5 μM. The photodynamic activity of each PS was evaluated by comparing the bacterial concentration of treated samples with that of LCs at each time point.

**Figure 5:**
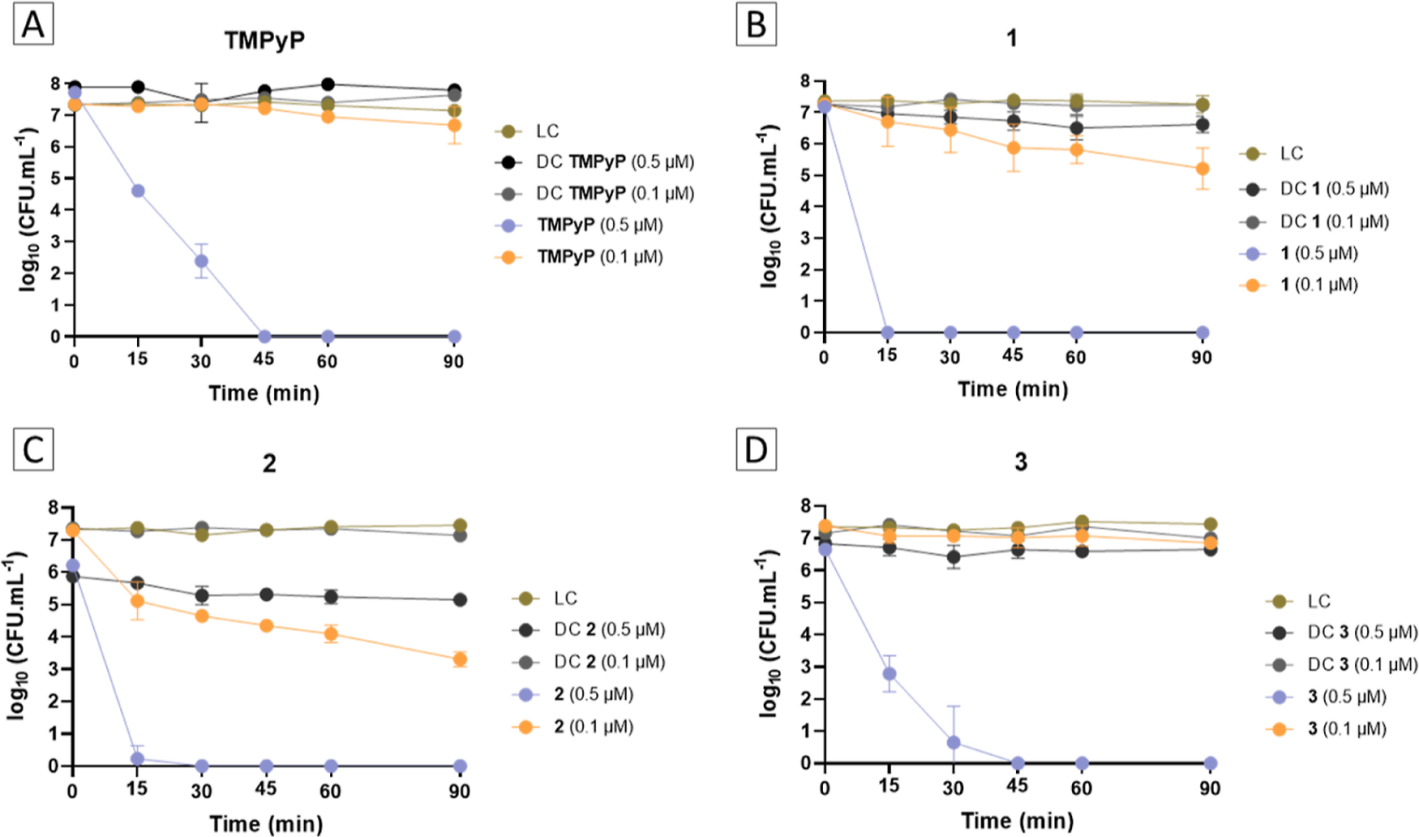
Photodynamic inactivation profile of MRSA promoted by porphyrin-based PS **TMPyP** (A), **1** (B), **2** (C) and **3** (D) at 0.5 and 0.1 μM and irradiated with white light at an irradiance of 25 mW cm^–2^. LC—light control; DC—dark control. Values are presented as the mean of 3 independent assays with two replicates each; the standard deviation is represented by the error bars. Lines just combine points.

The inactivation profile of MRSA in the presence of each porphyrin–triphenylphosphonium conjugate **1–3** and **TMPyP** shows that at a concentration of 0.5 μM all derivatives are able to photoinactivate MRSA to the detection limit of the method but required different irradiation times (Figure 5). For the reference **TMPyP** the detection limit was reached after 45 min of irradiation (decrease of 7.4 log_10_ cfu mL^–1^ in the bacterial viability) (ANOVA *p* < 0.05) (Figure 5A). A similar behavior was observed for the tetracationic conjugate **3**, reaching the detection limit of the methodology after the same 45 min of irradiation time (Figure 5D). However, PS **3** exhibits a slightly higher photoinactivation rate profile compared to **TMPyP**, with a bacterial concentration decrease of ∼6.6 log_10_ cfu mL^–1^ after 30 min, compared to ∼4.9 log_10_ cfu mL^–1^ exhibited by **TMPyP** (ANOVA *p* < 0.05). Notably, the highly charged conjugates **1** and **2** showed greater effectiveness, achieving the same inactivation but just after 15 min of irradiation, respectively (Figure 5B,C).

Impelled by these remarkable results the photodynamic activity of all the PS studied was also evaluated at a reduced concentration of 0.1 μM. In general, at this concentration a decrease in the photodynamic effectiveness of each porphyrin-based PS was observed (Figure 5A–D). In fact, at 0.1 μM both tetracationic PS **3** and **TMPyP** were ineffective in reducing the MRSA concentration (ANOVA *p* > 0.05) (Figure 5A,D). Once again, PS **1** and **2** demonstrated better photodynamic inactivation ability, with reductions of ∼2.0 and 4.1 log_10_ cfu mL^–1^ (ANOVA *p* < 0.05), respectively, after 90 min of irradiation (Figure 5B,C).

The results indicate that the various photodynamic activity profiles displayed by porphyrin-based PS in the photoinactivation of MRSA are strongly linked to the presence of triphenylphosphonium salt units within the porphyrin macrocycle, as well as the number of positive charges. Generally, the PS with a high number of positive charges led to a faster and more pronounced decline in the viability of the Gram-positive bacteria: PS **1** (+8) and **2** (+6) were the most efficient in the bacterium inactivation, followed by PS **3** (+4) and **TMPyP** (+4). These results align with the data reported in the literature, which suggests that an increase in the number of positive charges present in the porphyrinic structure typically enhances its affinity for bacterial cells due to increased favorable electrostatic interactions. Consequently, this leads to higher efficacy in aPDT.^17,45^ However, it must be highlighted, that the higher effectiveness of these conjugates compared to **TMPyP** may also stem from the presence of triphenylphosphonium groups. Prior studies have shown the potential of triphenylphosphonium units to boost the activity against Gram-positive bacterial strains by reducing the minimal inhibitory concentration compared to their nonsubstituted counterparts or quaternary ammonium compounds.^42,44^ This synergistic effect has been attributed to the substantial improvement of the hydrophobic surface area and dispersed charge distribution of triphenylphosphonium groups, along with its ability to alter the proton motive force.^42,44^

Recently, researchers have been exploring the potential of nonporphyrinic PS that have been modified with triphenylphosphonium units. In 2018, Bresolí-Obach and colleagues^45^ demonstrated that phenalenone and perylene derivatives, when modified with triphenylphosphonium moieties, showed enhanced activity against the Gram-positive bacteria *S. aureus* and *E. faecalis*. This enhanced activity was compared to their nonsubstituted counterparts, which did not exhibit any photodynamic activity against these bacteria.^45^ Another study emphasized the effectiveness of cyanine-based dye containing a triphenylphosphonium unit in photoinactivating *S. aureus*. This dye outperformed its carboxylate, sulfonyl and amino (primary, secondary and tertiary) counterparts. Furthermore, cyanine–triphenylphosphonium derivative showed the highest uptake value for *S. aureus* cells, indicating a strong affinity of this compound for the bacterial cell wall.^64^ This suggests that triphenylphosphonium moieties, with their large hydrophobic surface area and dispersed charge distribution, facilitate the adhesion of PS to biological membranes, causing their disruption and improving their antibacterial activity.^42,43,45^ In our study, bacterial uptake assays supported this trend, as the porphyrin–triphenylphosphonium conjugates **1–3** showed better adhesion to bacterial cells compared to the **TMPyP**.

It is important to note that both the LC and DC experiments showed that MRSA cell viability was not affected by light alone or by porphyrin–triphenylphosphonium conjugates **1** and **3**, nor by the reference **TMPyP** in the absence of light. This indicates that the reduction in bacteria was indeed due to the photodynamic effect. However, a noticeable toxicity was observed during the incubation period for PS **2** at a concentration of 0.5 μM, resulting in a reduction of 1.4 log_10_ cfu mL^–1^ (*p* < 0.05). This initial toxicity could be attributed to the triphenylphosphonium groups present in this PS.^44^ Nevertheless, it is important to mention that no toxicity was observed for PS **2** at the lowest concentration tested (0.1 μM). At this concentration, the PS was able to cause a bacterial reduction exceeding 3 log_10_ cfu mL^–1^, meeting the conditions necessary to be considered an effective antimicrobial agent.^65^

#### 3.2.3 Photodynamic Inactivation of *E. coli*

The effectiveness of porphyrin–triphenylphosphonium conjugates **1–3** in reducing the viability of a harmful Gram-negative bacterial strain was evaluated using the bioluminescent strain of *E. coli* as our experimental model. This particular strain serves as an excellent indicator of the effectiveness of the photodynamic process because the emitted light provides valuable insights into its metabolic activity. Additionally, using bioluminescence, the vitality of microorganisms is promptly assessed in real-time, presenting a faster and cost-effective alternative to traditional plating techniques.

**Figure 6:**
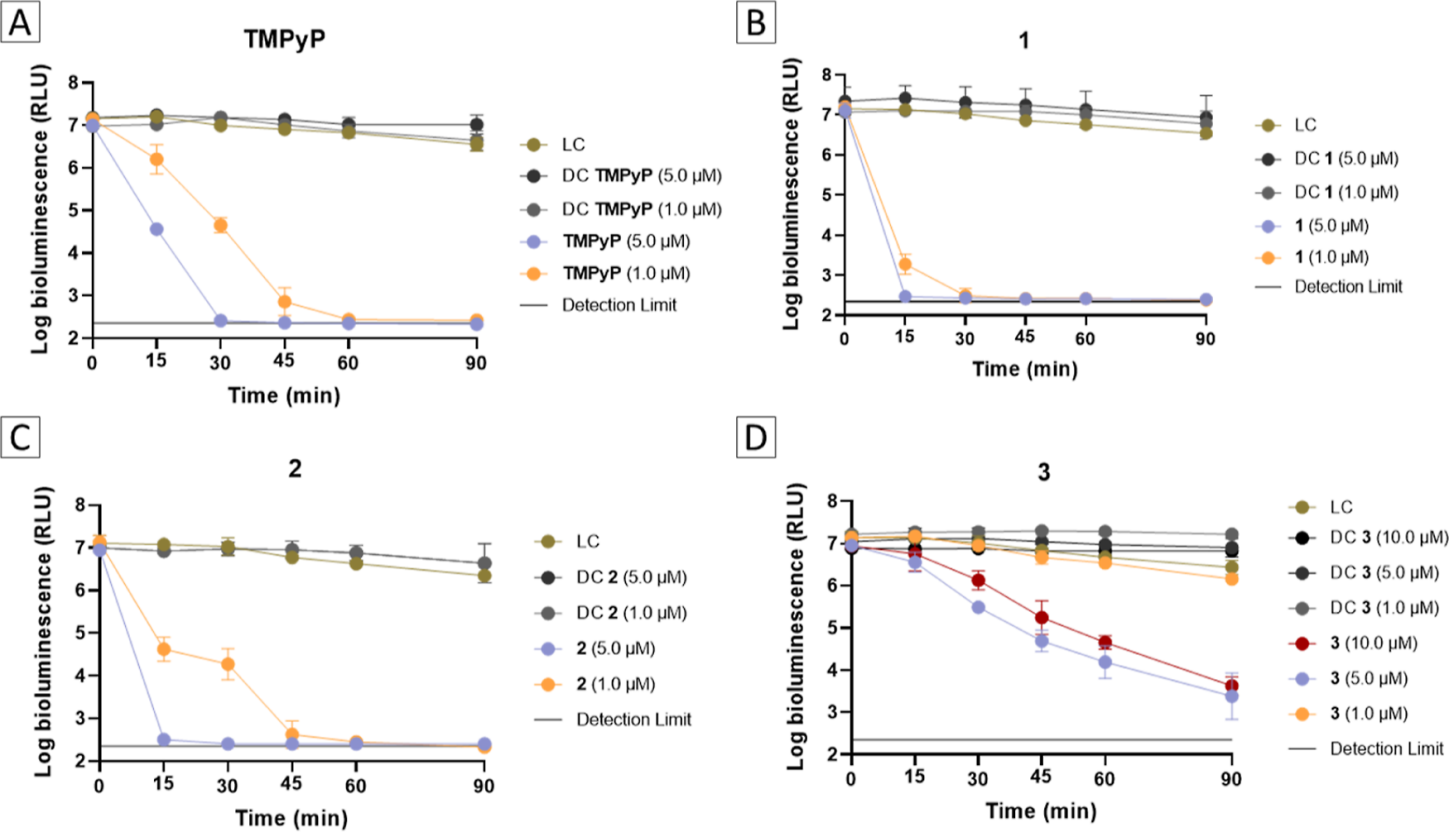
Photodynamic inactivation profile of bioluminescent *E. coli* promoted by porphyrin-based PS **TMPyP** (A), **1** (B), **2** (C) and **3** (D) at 1.0 and 5.0 μM and irradiated with white light at an irradiance of 25 mW cm^–2^. LC—light control; DC—dark control. Values are presented as the mean of 3 independent assays with two replicates each; the standard deviation is represented by the error bars. Lines just combine points.

Considering the reduced susceptibility of Gram-negative bacteria to aPDT, the photodynamic impact of the porphyrin–triphenylphosphonium conjugates and of **TMPyP** toward *E. coli* photoinactivation was tested at concentrations of 1.0 and 5.0 μM. The evaluation involved exposing the bacteria to white light (380–700 nm) at an irradiance of 25.0 mW cm^–2^ for 90 min in the presence of each porphyrinic PS. The outcomes, depicted in Figure 6, illustrate the effectiveness of the porphyrin–triphenylphosphonium derivatives **1–3** and the standard **TMPyP** in diminishing the viability of *E. coli*. The data obtained indicates that all PS, apart from compound **3**, were effective in reducing the viability of *E. coli* until the luminometer’s detection limit and the photoinactivation profiles were dependent on the PS̀s concentrations and irradiation times.

Similar to the observations made in the *S. aureus* photoinactivation studies, the porphyrin–triphenylphosphonium conjugate **1** exhibited the greatest effectiveness in the photodynamic inactivation of *E. coli*. Following suit were PS **2**, **TMPyP**, and **3** in descending order of efficacy.

In the case of the tetracationic standard **TMPyP** a significant reduction in the bioluminescence of *E. coli* (ca 4.6 log_10_ URL; *p* < 0.05) was observed at both 5.0 and 1.0 μM concentrations after 30 and 60 min of treatment, respectively (Figure 6A).

The top-performing conjugates **1** and **2** at a concentration of 5.0 μM, were able to induce a rapid decrease in the viability of bioluminescent *E. coli*, reaching the detection limit of the methodology after 15 min of irradiation, resulting in a reduction of 4.6 log_10_ URL (*p* < 0.05) (Figure 6B,C). However, a distinct behavior was observed when the PS concentration was reduced to 1.0 μM. At this concentration, PS **1** was able to achieve the detection limit of the methodology after 30 min of irradiation, while PS **2** required 45 min of irradiation to achieve a similar effectiveness. When the PS concentration was reduced to 1.0 μM, PS **2** exhibited a behavior corresponding to that presented by **TMPyP**, with a similar bacterial photoinactivation rate. Nevertheless, despite the significant reduction on its concentration, PS **1** was able to induce a reduction of 3.9 log_10_ (*p* < 0.05) after just 15 min of irradiation (Figure 6A–C).

In comparison, PS **3** proved to be the least efficient compound in the photoinactivation of the bioluminescent *E. coli* bacterium. At the lowest concentration of 1.0 μM, it did not lead to a significant decrease in bacterial viability (*p* > 0.05). However, at the concentration of 5.0 μM, PS **3** was able to reduce *E. coli* viability by approximately 3.1 log_10_ URL (*p* < 0.05) after 90 min of irradiation (Figure 6D). Due to the lower photoinactivation capacity demonstrated by conjugate **3** when compared to its counterparts **1** and **2**, its efficiency was further examined at 10.0 μM, without any positive outcome when compared with the results obtained at 5.0 μM (*p* > 0.05) (Figure 6D).

Despite porphyrin **3** having the same number of positive charges as **TMPyP** and superior affinity for bacterial cells, it exhibited reduced photodynamic efficacy. This can be related to its reduced solubility in PBS and tendency to aggregate resulting in a decrease of ^1^O_2_ production.^66,67^ On the other hand, porphyrin–triphenylphosphonium conjugates **1** and **2** showed higher stability in solution, under dark and irradiation conditions, which can contribute to their high efficacy.

Bresolí-Obach et al. observed the ineffectiveness of a phenalenone triphenylphosphonium-modified PS against Gram-negative bacteria; these results were mainly attributed to their high hydrophobicity, which hampers the penetration of the lipopolysaccharide barrier present in Gram-negative bacteria.^45^ Contrary to this, in our study, we found that, in fact, all PS were effective in reducing the viability of *E. coli*. Noting that PS **3** demonstrated less efficiency than PS **1** and **2** which can be associated with its more reduced solubility and tendency to aggregate than the other two conjugates decreasing its photodynamic activity against the Gram-negative bacteria.

Comparing the results obtained with all porphyrins in *S. aureus* and *E. coli*, a relationship was observed between the concentration of PS used and the bacteria tested. Higher concentrations of PS were required for the efficient inactivation of *E. coli*, reinforcing the lower susceptibility of Gram-negative bacteria compared to Gram-positive bacteria, caused by differences in their cell walls.^68^ However, the porphyrin–triphenylphosphonium conjugates evaluated in this study proved to be effective PS against both bacterial models. Considering that the observed inactivation was greater than 3 log cfu mL^–1^ (99.9% reduction), the evaluated compounds can be considered as efficient bactericidal agents.^65^ In fact, the minimum bactericidal concentration (MBC), that resulted in at least a 3-log reduction in bacterial count, was achieved for *S. aureus* with 0.5 μM for all tested PS, with variations in required irradiation times (up to 45 min). While for *E. coli*, the MBC was 5.0 μM for PS **3** and 1.0 μM for the remaining PS, also with variations in the required irradiation time (up to 90 min). Furthermore, it is noteworthy that these porphyrin–triphenylphosphonium conjugates allowed a significant improvement in the efficacy of aPDT compared to the standard porphyrin **TMPyP**, allowing a reduction in irradiation time of more than 50%.

It is important to note that neither the light conditions used affected the bacterial cells viability, nor did the PS presented noticeable toxicity in the dark even at the highest concentration tested.

The results obtained from the aPDT studies against both bacterial strains highlighted the relevance to coupling triphenylphosphonium salt moieties to porphyrin macrocycle, leading to porphyrin-based PS with improved photodynamic activity. The presence of triphenylphosphonium units on the porphyrinic macrocycle clearly promotes better interaction of the PS with the bacterial membrane. However, the proximity of two positive charges, from the pyridinium and triphenylphosphonium units in each porphyrinic *meso* position is a key structural feature to enhance the PS activity of the porphyrin-based PS leading to a faster bacterial photoinactivation. This makes PS **1** a remarkable PS with high effectiveness in the photodynamic inactivation of both Gram-positive and Gram-negative bacteria, overcoming the performance of all the other studied PS, including the standard **TMPyP**.

## 4 Conclusions

In this study, were obtained the porphyrin–triphenylphosphonium conjugates **1–3** via *N*-alkylation of pyridyl units or nucleophilic substitution reactions using the adequate scaffolds. The synthetic approaches provided cationic porphyrin-based PS with positive charges ranging between 4 and 8. This fact enabled their ability to generate singlet oxygen to be modulated, as well as their stability and solubility in aqueous medium. By conjugating an increasing number of triphenylphosphonium moieties at the *meso* positions of the porphyrin macrocycle, the capability of the compounds to generate singlet oxygen was enhanced and their photo(stability). As a result, were obtained stable compounds with high potential for use in photodynamic processes.

Biological assays conducted against Gram-positive *S. aureus* and Gram-negative *E. coli* revealed that porphyrin–triphenylphosphonium conjugates **1** and **2** exhibited significantly higher effectiveness than the reference **TMPyP**, in photoinactivating both bacteria. Their better photodynamic activity is attributed to the presence of a greater number of positive charges and triphenylphosphonium units, which promoted better adhesion of the PS to bacterial cells. However, although porphyrin **3** demonstrated similar performance than **TMPyP** toward *S. aureus*, its inferior outcome against *E. coli* can be ascribed to its lower stability and greater tendency to aggregate.

The use of porphyrin–triphenylphosphonium conjugates **1** and **2** allowed reaching the full photoinactivation of bacteria, with a 66% reduction in irradiation time for *S. aureus* and a 50% reduction for *E. coli* compared to the time required for **TMPyP**. This achievement is noteworthy, highlighting the considerable efficacy of combining porphyrinic macrocycles with triphenylphosphonium units to modulate and enhance PS activity against bacterial strains.

In summary, the free base porphyrin–triphenylphosphonium conjugates (**1**, **2** and **3**) evaluated in this study have shown to be efficient bactericidal agents. Moreover, this research provides the groundwork for the development of new PS agents, opening new avenues in both porphyrin chemistry and aPDT.

## Supporting Information Available

NMR and HRMS-ESI(+)] spectra (PDF)
