# Supplementary material for: Can Porphyrin–Triphenylphosphonium Conjugates Enhance the Photosensitizer Performance Toward Bacterial Strains?
Source: ACS Appl Bio Mater. 2024 Jul 15;7(8):5541–52. doi: 10.1021/acsabm.4c00659 (PMC11337165; doi:10.1021/acsabm.4c00659)
Supplement: Supplementary file 1 — mt4c00659_si_001.pdf [file mt4c00659_si_001.pdf]

Supporting information

Can porphyrin-triphenylphosphonium conjugates enhance the photosensitizer performance towards bacterial strains?

Inês Chaves[a], Filipe M. P. Morais[b], Cátia Vieira[a], Maria Bartolomeu[a], M. Amparo F. Faustino[b], M. Graça P. M. S. Neves[b], Adelaide Almeida[a,*], Nuno M. M. Moura[b,*]

[a] CESAM, Department of Biology, University of Aveiro, 3810-193 Aveiro, Portugal

[b] LAQV-REQUIMTE, Department of Chemistry, University of Aveiro, 3810-193 Aveiro, Portugal

Corresponding authors: aalmeida@ua.pt (A.A.); nmoura@ua.pt (N.M.M.M.)

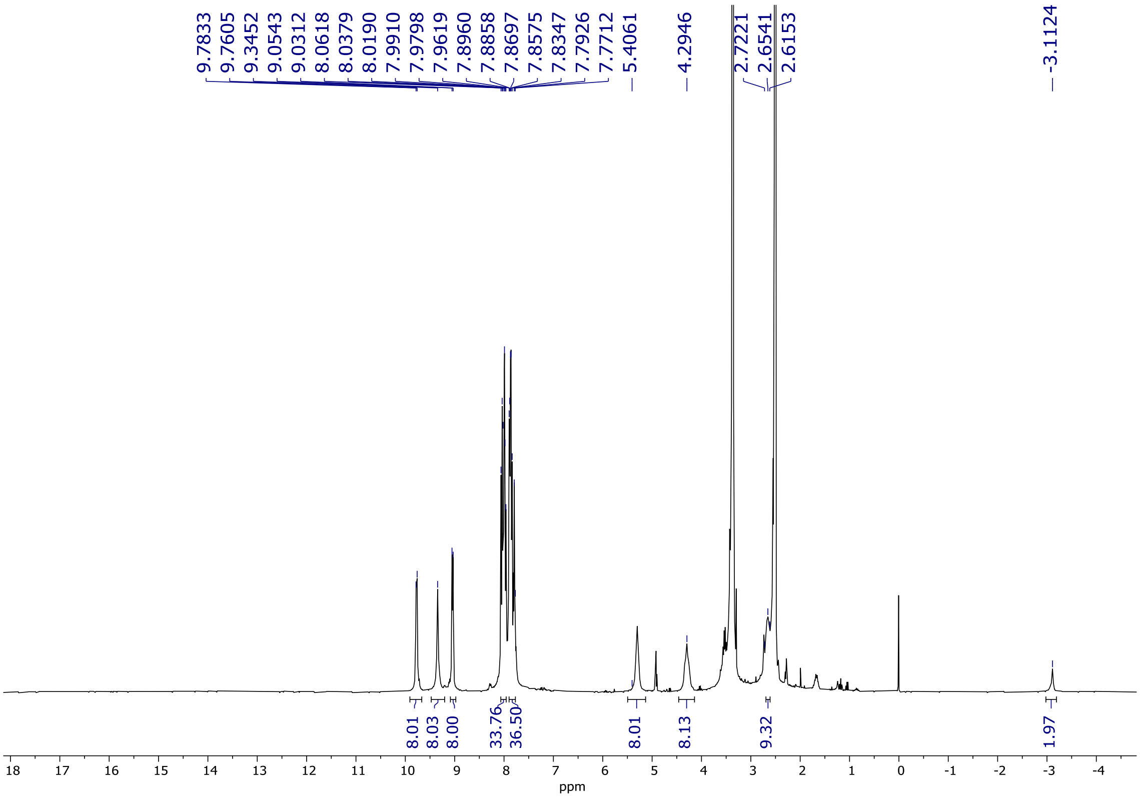

Figure S1. ^{1}H NMR spectrum of compound **1** in DMSO-d_6.

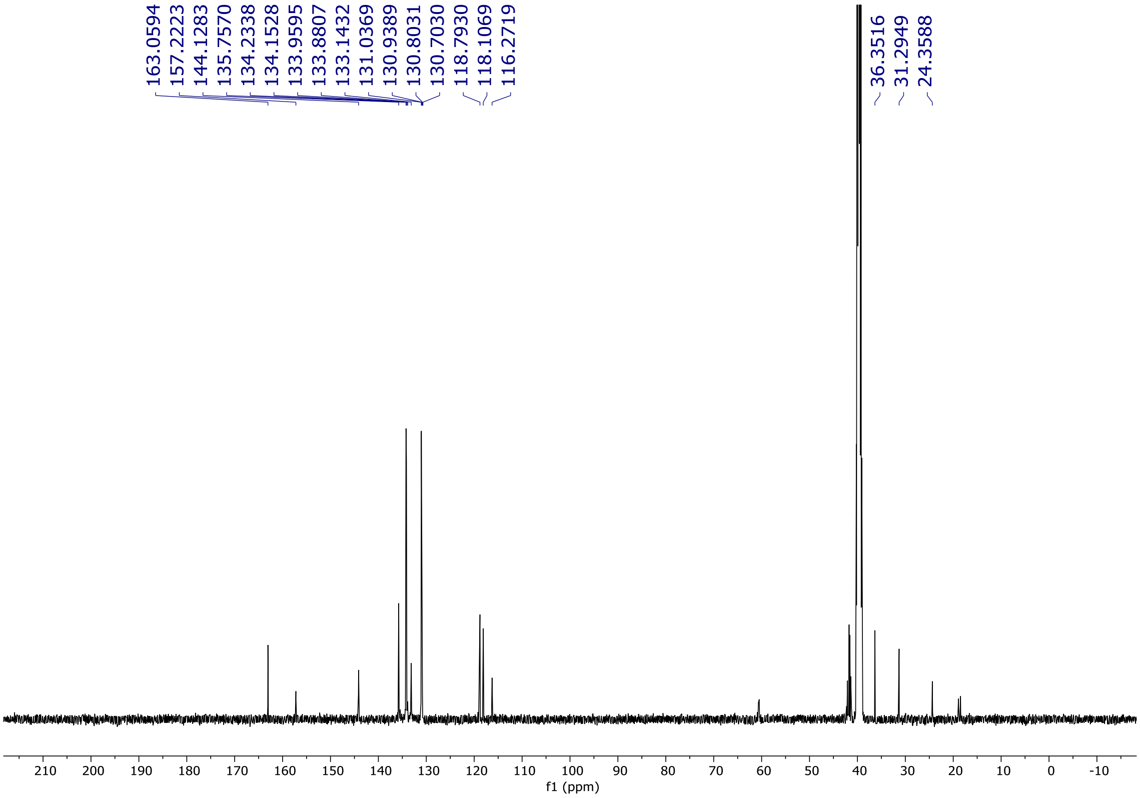

Figure S2. ^{13}C NMR spectrum of compound **1** in DMSO-d_6.

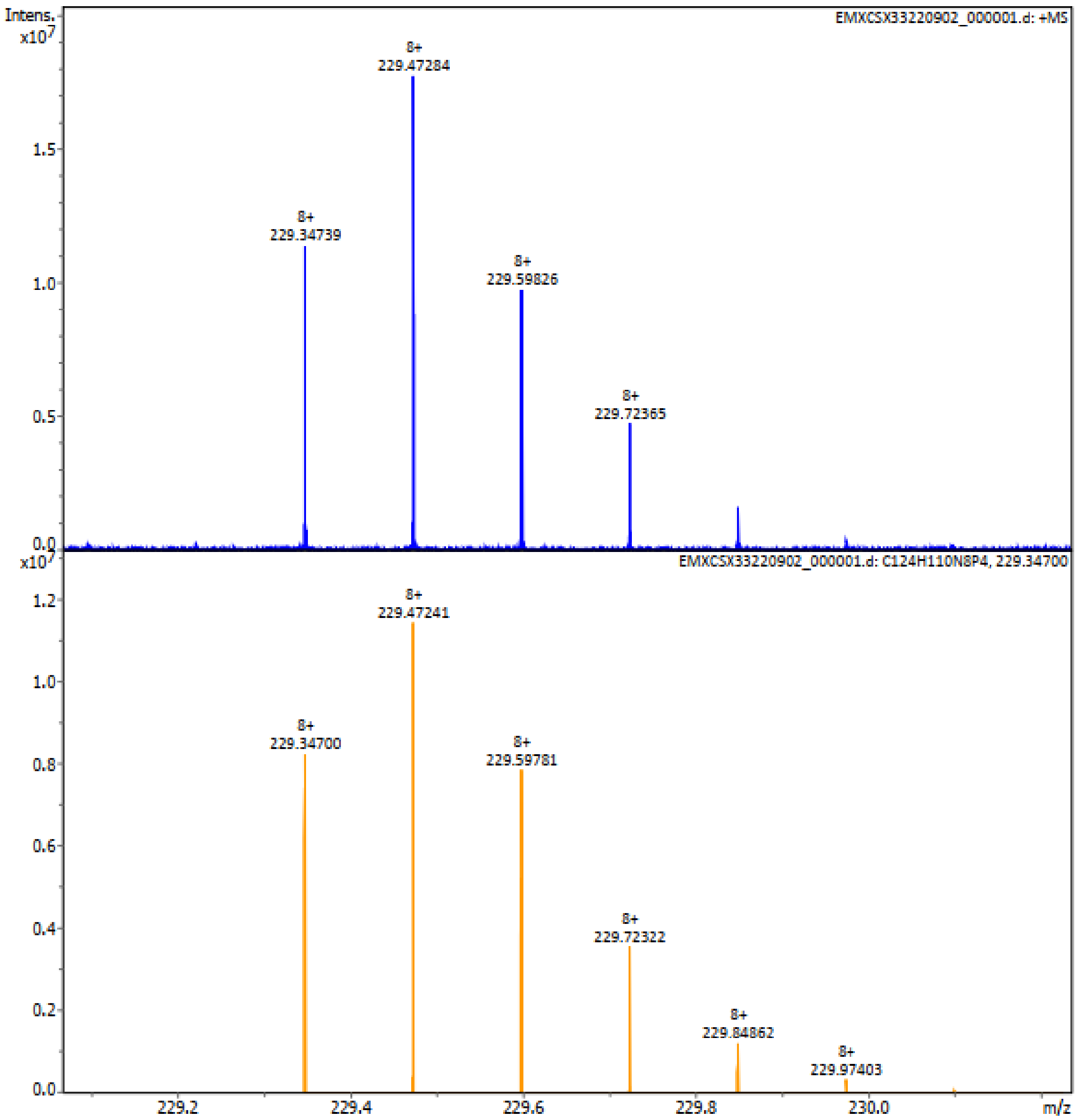

Mass Spectrum Molecular Formula Report

Meas. m/z	#	Ion Formula	Score	m/z	err [ppm]	Mean err [ppm]	mSigma	rdb	e⁻ Conf	N-Rule
229.347389	1	C124H110N8P4	100.00	229.347001	-1.7	-1.1	63.7	84.0	even	ok

Figure S3. HRMS spectrum of compound **1** (top) observed experimental pattern of the M^{8+} ions; (middle) calculated isotopic pattern of the M^{8+} ions; (bottom) molecular formula report.

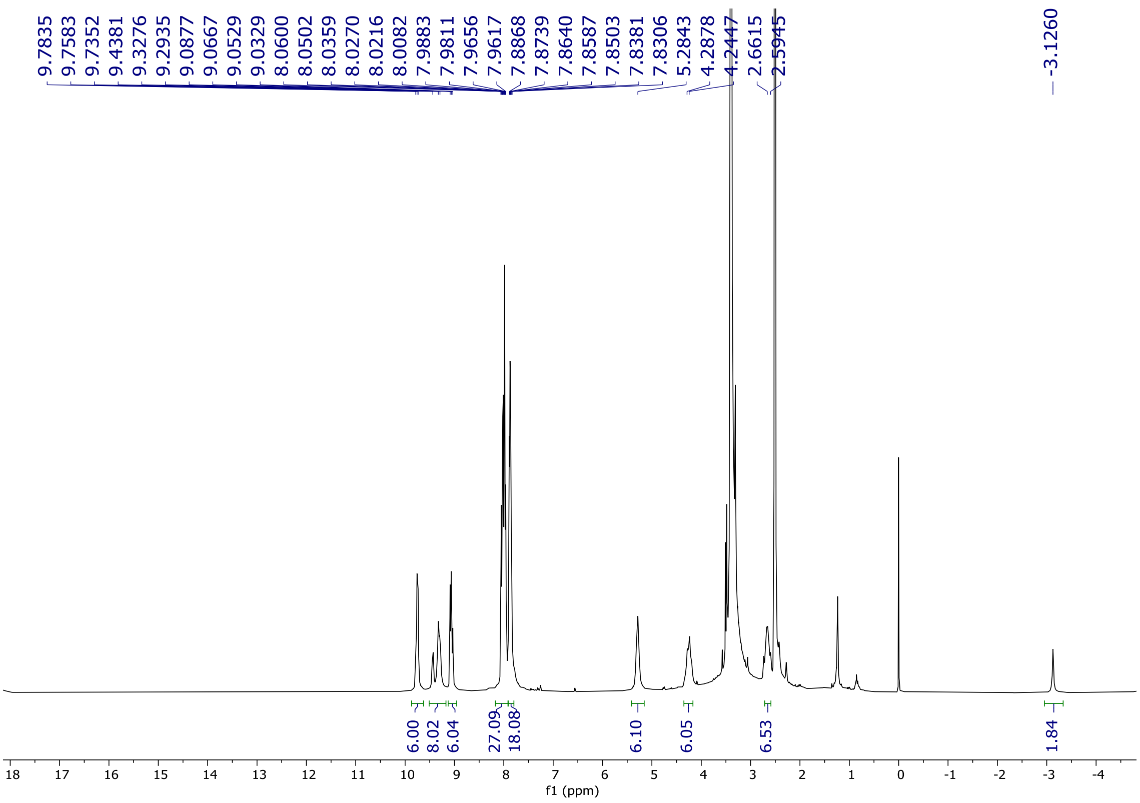

Figure S4. ^{1}H NMR spectrum of compound **2** in DMSO-d_6.

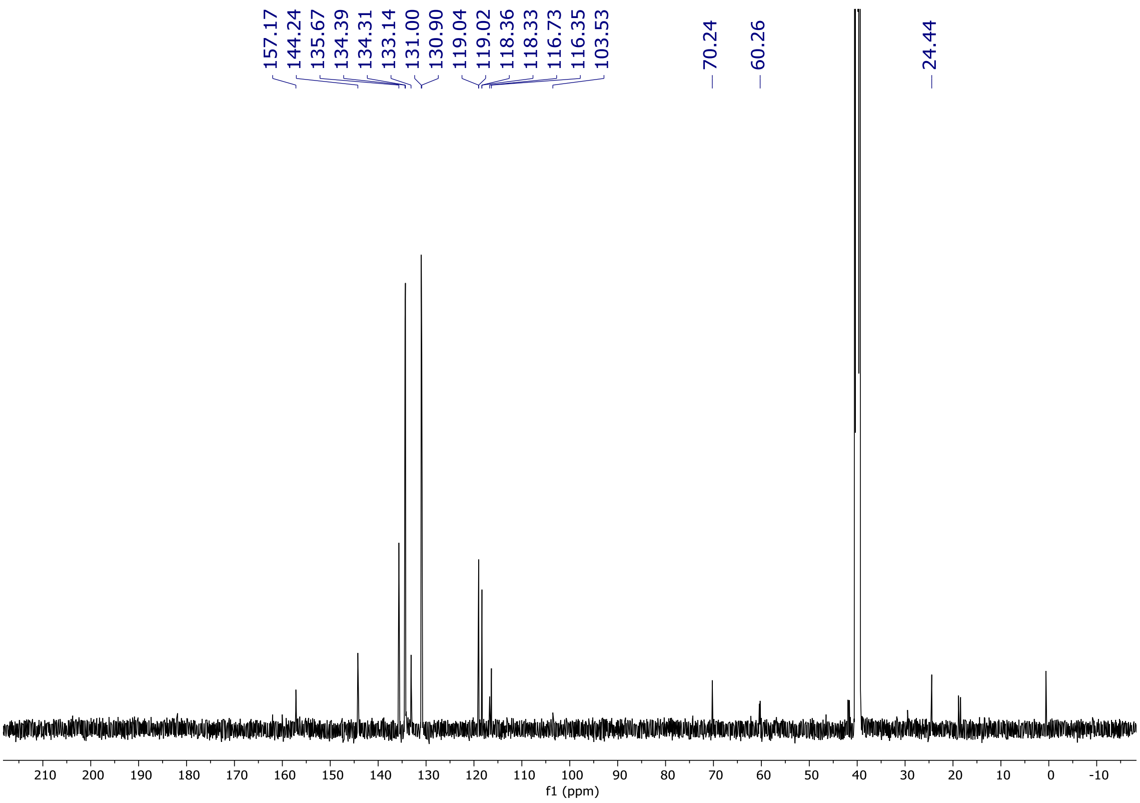

Figure S5. ^{13}C NMR spectrum of compound **2** in DMSO-d_6.

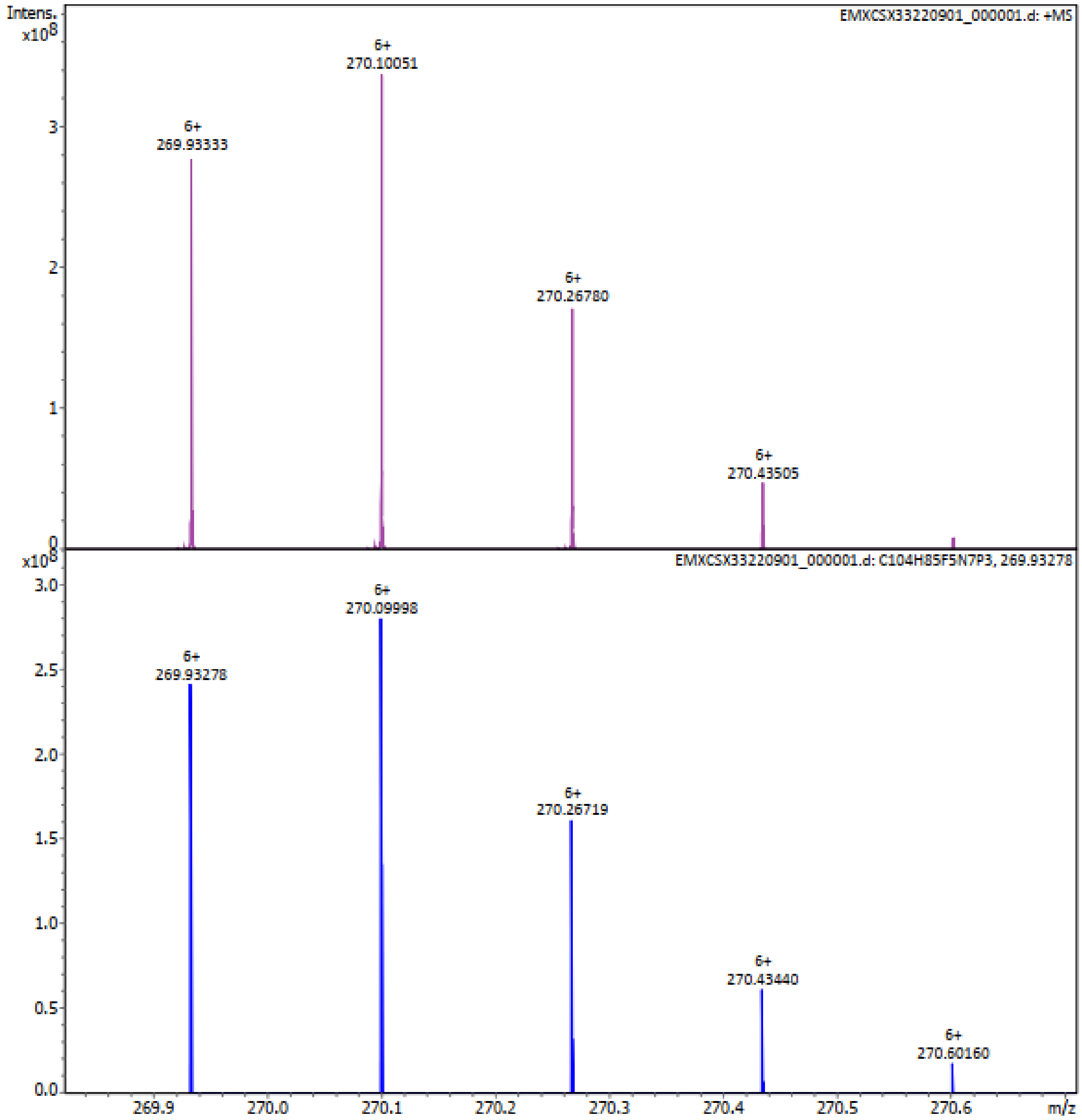

Mass Spectrum Molecular Formula Report

Meas. m/z	#	Ion Formula	Score	m/z	err [ppm]	Mean err [ppm]	mSigma	rdb	e⁻ Conf	N-Rule
269.933332	1	C104H85F5N7P3	100.00	269.932776	-2.1	-1.2	38.7	71.0	even	ok

Figure S6. HRMS spectrum of compound **2** (top) observed experimental pattern of the M^{6+} ions; (middle) calculated isotopic pattern of the M^{6+} ions; (bottom) molecular formula report.

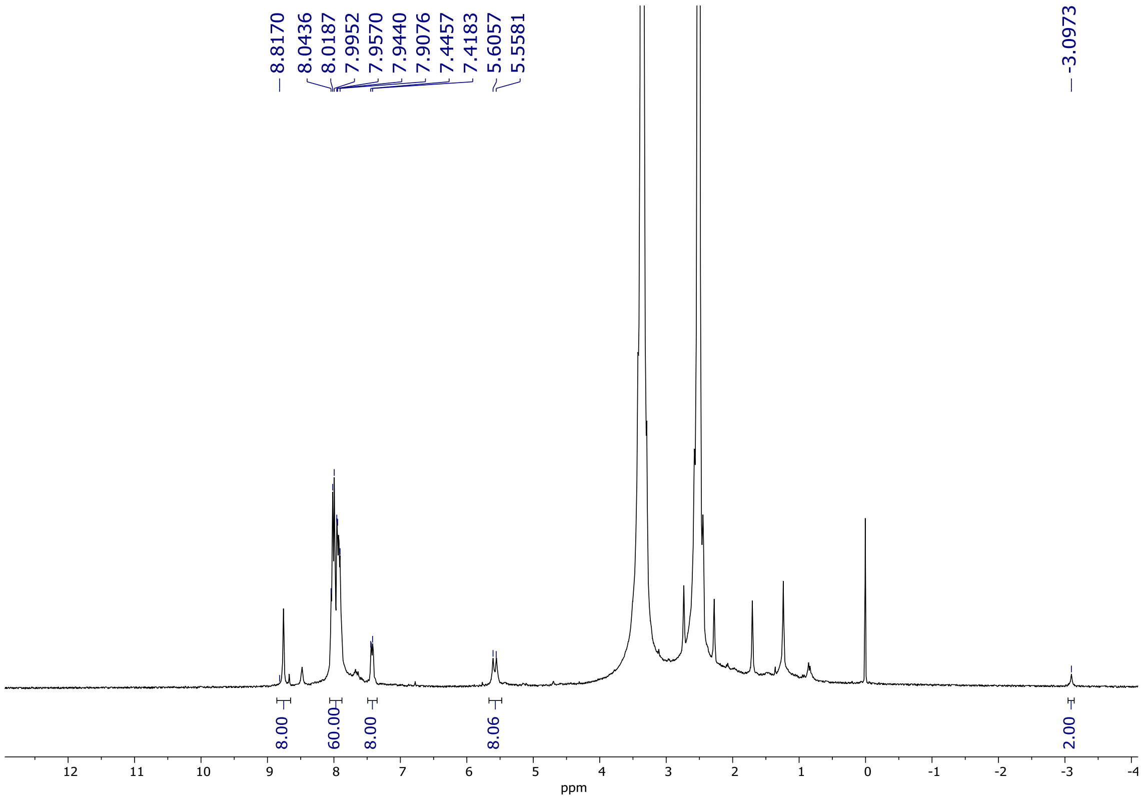

Figure S7. ^{1}H NMR spectrum of compound **3** in DMSO-d_6.

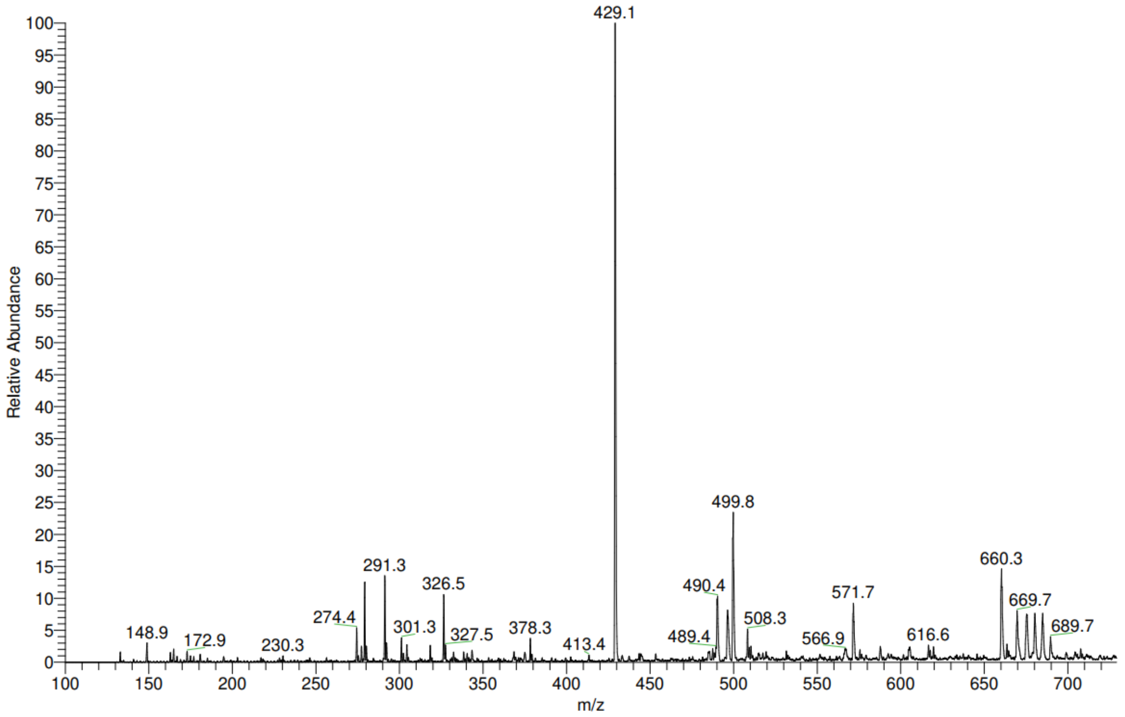

Figure S8. MS-ESI(+) spectrum of compound **3**.

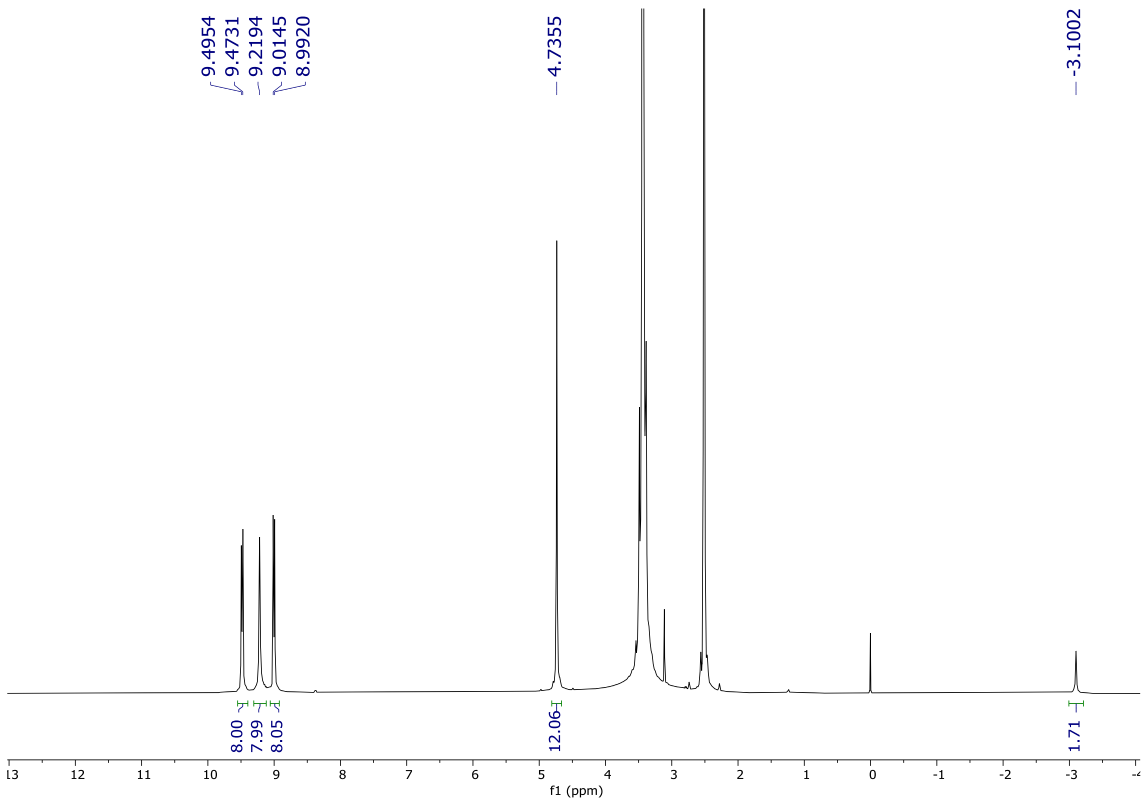

Figure S9. ^{1}H NMR spectrum of **TMPyP** in DMSO-d_6.

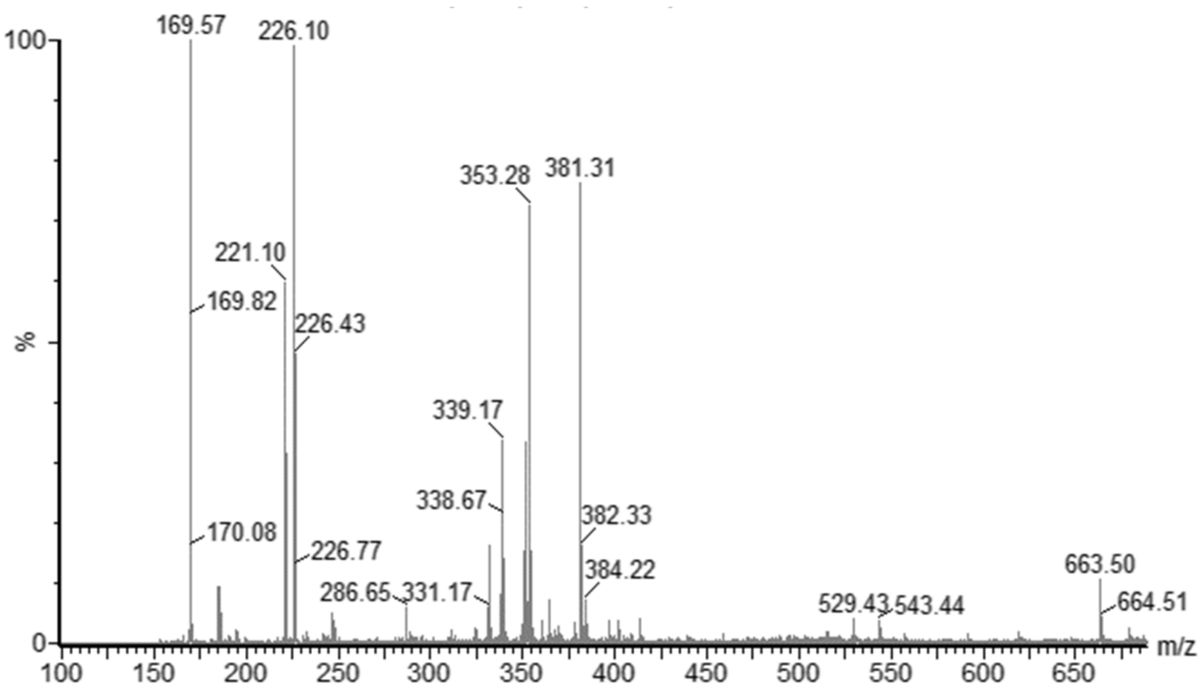

Figure S10. MS-ESI(+) spectrum of **TMPyP**.
